# Association of Postoperative Drainage Strategy with Persistent Urinary Leakage and Clinical Outcomes Following Percutaneous Nephrolithotomy

**DOI:** 10.3390/medicina62091759

**Published:** 2026-09-12

**Authors:** Anıl Eker, Kemal Züngün, Muhammet Bilal Nart, Muhammet Halil Dağaşan, Serkan Yarımoğlu, Murat Şahan, İbrahim Halil Bozkurt, Tansu Değirmenci

**Affiliations:** 1 Department of Urology, Izmir City Hospital, Izmir 35530, Türkiye; m_b_nart@hotmail.com (M.B.N.); muratsahan87@hotmail.com (M.Ş.); 2 Department of Urology, Izmir City Hospital, University of Health Sciences, Izmir 35530, Türkiye; kemalzungun@gmail.com (K.Z.); mhalildagasan@hotmail.com (M.H.D.); 3 Department of Urology, Izmir Faculty of Medicine, University of Health Sciences, Izmir 35530, Türkiye; serkanyarimoglu@gmail.com (S.Y.); ihalilbozkurt@yahoo.com (İ.H.B.); tansudegirmenci@hotmail.com (T.D.)

**Keywords:** percutaneous nephrolithotomy, double-J stent, ureteral catheter, urinary leakage, postoperative complications

## Abstract

*Background and Objectives:* Persistent postoperative urinary leakage may delay recovery after percutaneous nephrolithotomy (PCNL). This study evaluated the association between postoperative drainage strategy, persistent urinary leakage, and clinical outcomes following prone PCNL. *Materials and Methods:* This retrospective, non-randomized cohort analysis included 606 consecutive eligible patients undergoing prone PCNL between 2015 and 2026 from a prospectively maintained institutional database. Patients received either an external ureteral catheter (*n* = 529) or primary antegrade double-J (DJ) stent (*n* = 77). Perioperative outcomes were compared, and factors associated with persistent urinary leakage were evaluated in the ureteral-catheter group. *Results:* Operative and nephroscopy times were longer in the DJ-stent group (110.4 ± 47.2 vs. 100.6 ± 37.9 min, *p* = 0.041; 50.0 vs. 40.0 min, *p* = 0.002). Persistent urinary leakage occurred in 39 patients (7.4%) with ureteral catheters and in none with primary DJ stents (*p* = 0.006). After stabilized IPTW, the risk difference for primary DJ stenting versus ureteral-catheter drainage was −7.45 percentage points (95% bootstrap CI, −9.77 to −5.33). The distributions of nephrostomy tube removal time and hospital stay differed between the drainage groups (DJ stent vs. ureteral catheter: 2.0 [IQR, 2.0–2.0] vs. 2.0 [IQR, 2.0–2.5] days, *p* = 0.028; and 3.0 [IQR, 2.0–3.0] vs. 3.0 [IQR, 3.0–4.0] days, *p* = 0.004, respectively), although the corresponding Hodges–Lehmann estimated differences were small (0 days [95% CI, 0 to 0] and 0 days [95% CI, 0 to 1], respectively). In multivariable analysis, residual stone presence was associated with higher odds of persistent urinary leakage (adjusted OR 2.16, 95% CI 1.09–4.28; *p* = 0.027). *Conclusions:* Primary antegrade DJ stenting was associated with lower urinary leakage rates. Although the distributions of nephrostomy tube removal time and hospital stay also favored the DJ-stent group, the absolute between-group differences were small. Residual stone presence remained independently associated with higher odds of persistent urinary leakage.

## 1. Introduction

Urolithiasis is a common health problem worldwide and represents a significant source of morbidity and healthcare burden. The prevalence of urinary stone disease has increased over recent decades due to changes in dietary habits, obesity, metabolic disorders, and environmental factors [1]. Percutaneous nephrolithotomy (PCNL) is currently considered the standard treatment modality for renal stones larger than 2 cm because of its high stone-free rates and acceptable safety profile [2].

Traditionally, PCNL has been performed in the prone position with placement of a nephrostomy tube at the end of the procedure in order to provide urinary drainage, tamponade the nephrostomy tract, and facilitate secondary access if necessary. However, advances in endourological technology and increasing surgical experience have led to the development of alternative approaches such as tubeless PCNL, supine PCNL and mini-PCNL, which are increasingly utilized in selected patients [3,4,5]. Despite being a commonly performed endourological procedure, considerable variability still exists among centers and surgeons regarding both the operative technique and postoperative drainage strategies used during PCNL.

Although completely tubeless approaches without a nephrostomy tube or ureteral drainage have gained increasing interest, conventional PCNL with nephrostomy tube placement accompanied by ureteral drainage remains a commonly used postoperative drainage approach. In these patients, postoperative urinary drainage is generally achieved either by placement of an external ureteral catheter or an internal DJ stent [3,6]. However, there is currently no clear consensus regarding which drainage method should be preferred, and the choice is largely based on surgeon preference and institutional experience. Furthermore, previous studies evaluating postoperative urinary leakage have predominantly focused on patients managed with external ureteral drainage, whereas data including patients undergoing primary antegrade double-J (DJ) stent placement remain limited [7].

The present study aimed to evaluate the association between postoperative drainage strategy and perioperative and postoperative clinical outcomes following prone PCNL. In addition, we investigated the relationship between different drainage methods and persistent postoperative urinary leakage while identifying independent factors associated with its development in a large retrospective cohort of patients undergoing conventional PCNL.

## 2. Materials and Methods

This retrospective cohort study was conducted using a prospectively maintained institutional database of consecutive patients who underwent prone PCNL between January 2015 and January 2026 at a tertiary referral center. Perioperative and intraoperative data were prospectively and systematically recorded using a standardized institutional data collection form completed at the time of surgery.

After obtaining approval from the institutional ethics committee, a total of 775 patients who underwent prone PCNL were assessed for eligibility. After exclusion of 169 patients according to the predefined exclusion criteria, 606 patients were included in the final analysis.

**Figure 1:**
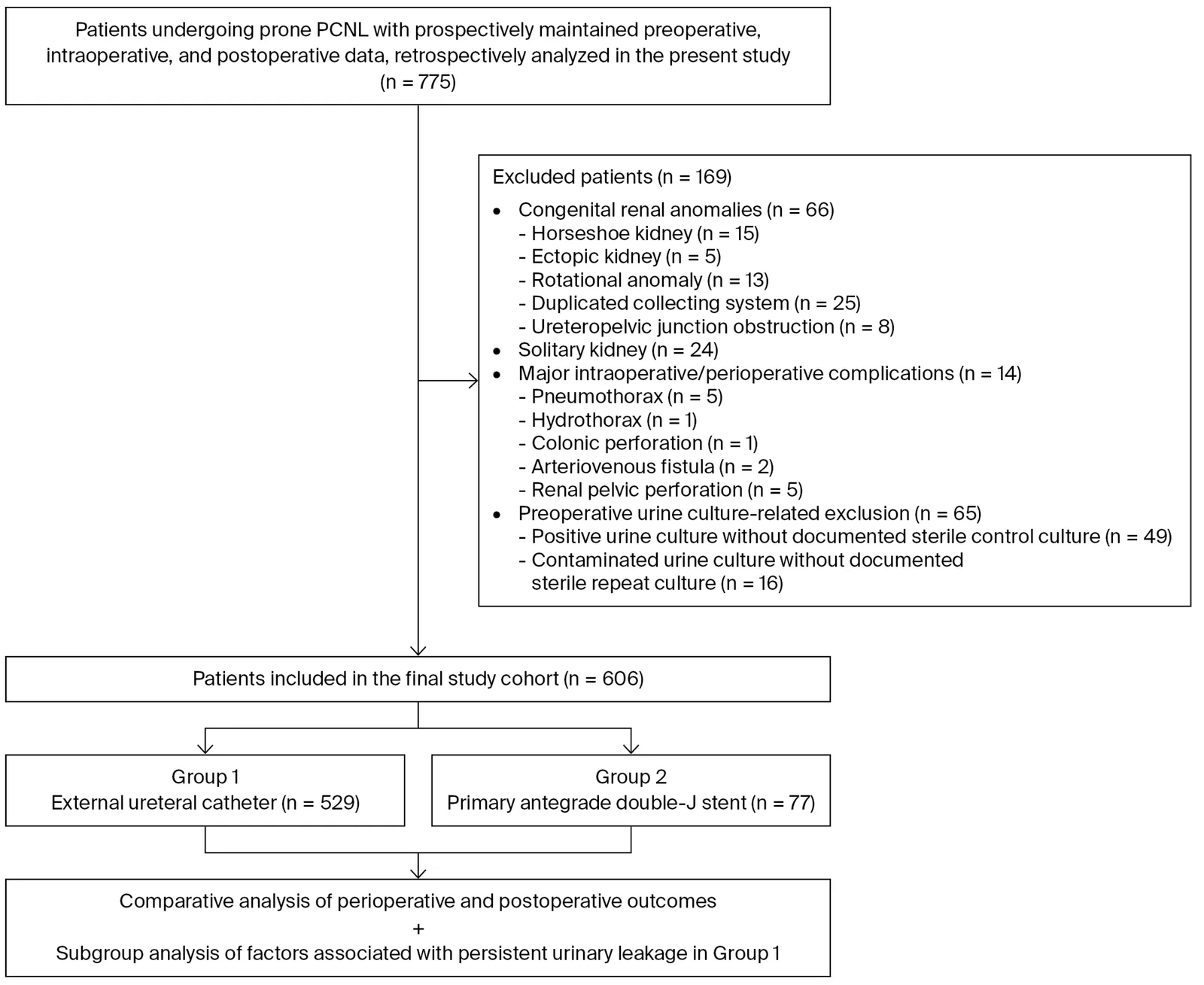
Patient selection flowchart.

Patients who experienced major intraoperative or perioperative complications requiring substantial deviation from the standard postoperative management pathway, including pneumothorax, hydrothorax, colonic perforation, arteriovenous fistula, and renal pelvic perforation, were excluded from the study. Patients with a solitary kidney or congenital renal anomalies were also excluded. According to our institutional protocol, all patients underwent preoperative urine culture assessment. Patients with a positive urine culture received culture-specific antibiotic therapy, and those with a documented sterile control culture before surgery were eligible for inclusion. Patients with a positive urine culture who underwent PCNL under culture-directed perioperative antibiotic coverage without documentation of a sterile control culture, as well as patients with contaminated urine cultures who underwent surgery under broad-spectrum third-generation cephalosporin prophylaxis without a documented sterile repeat culture, were excluded from the study. The detailed patient selection process and reasons for exclusion are presented in Figure 1.

Demographic characteristics, stone-related parameters, intraoperative findings, and postoperative outcomes were recorded for all patients. Perioperative variables included operative time, nephroscopy duration, fluoroscopy duration, irrigation fluid volume, puncture site, access calyx, number of access tracts, nephrostomy tube removal time, and length of hospital stay. Nephroscopy time was defined as the interval between successful entry into the renal collecting system and completion of the procedure. Nephroscopy time included the end-of-procedure drainage placement stage and therefore included the time required for antegrade DJ stent placement when this drainage strategy was used.

Residual stone status used in the analyses of persistent urinary leakage was determined using the perioperative assessment, including fluoroscopic imaging and direct nephroscopic visualization at the end of the procedure, together with plain kidney–ureter–bladder radiography obtained on the morning of the first postoperative day. Residual fragments smaller than 4 mm were not categorized as residual stones in these analyses and were considered consistent with surgical success. Postoperative residual fragments were further assessed by computed tomography performed during the first postoperative month. First-month CT findings were not used as predictor variables in the urinary leakage analyses and were assessed separately only for postoperative evaluation of surgical success.

Stone burden was calculated in square millimeters for all patients using the formula length × width × π × 0.25, where π was accepted as 3.14 [8]. In patients with multiple renal stones, each stone burden was calculated separately, and the total stone burden was determined as the sum of all individual stone areas.

The primary outcome of the study was persistent urinary leakage following nephrostomy tube removal. Secondary exploratory outcomes included intraoperative procedural parameters, including operative time, nephroscopy time, fluoroscopy time, and irrigation fluid volume, as well as postoperative outcomes including time to nephrostomy tube removal, length of hospital stay, infectious complications, blood transfusion, and other postoperative complications. Because no formal adjustment for multiple comparisons was performed, analyses of secondary outcomes were considered exploratory.

### 2.1. Surgical Technique

All procedures were performed under general anesthesia. Initially, a 5 F ureteral catheter was inserted into the collecting system of the stone-bearing kidney under cystoscopic guidance and fixed to a Foley catheter. Subsequently, patients were repositioned into the prone position. Percutaneous renal access was obtained under fluoroscopic guidance using an 18-G access needle. Tract dilation was performed using Amplatz dilators up to 30 F caliber, and a 26-F rigid nephroscope was used during the procedure. Stone fragmentation was achieved using a pneumatic lithotripter (Vibrolith; Elmed, Ankara, Turkey), and stone fragments were extracted when necessary.

The choice of postoperative drainage method was not standardized during the study period and was based on the operating surgeon’s individual preference and intraoperative judgment, without predefined institutional selection criteria. Both external ureteral catheterization and primary antegrade double-J stenting were routinely used at our institution. Accordingly, after completion of stone fragmentation, the existing external ureteral catheter was either retained or removed and replaced with a 4.8-F antegrade double-J stent advanced over a guidewire under fluoroscopic guidance. Correct stent positioning was confirmed fluoroscopically, and a 14-F nephrostomy tube was routinely placed in all patients.

Postoperatively, all patients underwent daily hemoglobin monitoring. On the morning of the first postoperative day, a plain urinary system radiograph (kidney–ureter–bladder radiography) was obtained in all patients to evaluate residual stone fragments and, when applicable, to confirm the position of the DJ stent. Subsequently, the nephrostomy tube was clamped in patients without fever, significant hematuria, or other major complications. Patients were subsequently monitored for flank pain suggestive of urinary obstruction secondary to inadequate drainage between the kidney and bladder. In cases of severe pain, the nephrostomy tube was reopened to re-establish drainage, and a repeat clamping trial was performed approximately 8 h later. In the absence of severe pain, obstruction, or impaired urinary drainage, the nephrostomy tube was removed. Following nephrostomy tube removal, patients were closely monitored for symptoms and signs of urinary leakage.

Following nephrostomy tube removal, all patients were monitored according to the same standardized ward protocol, including hourly assessment of the nephrostomy-site dressing by a resident physician. All patients remained hospitalized for more than 24 h after nephrostomy tube removal. In patients without ongoing urinary leakage, the Foley catheter and, when present, the external ureteral catheter were routinely removed 8–12 h after nephrostomy tube removal. If urinary leakage persisted during this interval, catheter removal was deferred until the leakage had resolved. Thus, the 8–12-h interval represented the standard postoperative protocol in patients without ongoing leakage, whereas removal was delayed when leakage persisted. Post-nephrostomy tract urinary leakage was defined as persistent wetting of the dressing after nephrostomy tube removal, either reported by the patient or documented during these hourly assessments. No biochemical confirmation of the urinary origin of the dressing fluid was performed. The onset of leakage was recognized at the first observed or reported dressing wetting, whereas resolution was defined as confirmation of a dry dressing by the treating physician. Urinary leakage persisting for more than 24 h after nephrostomy tube removal was classified as persistent urinary leakage. The volume of leakage was not quantitatively measured, and the exact duration beyond the 24-h threshold was not systematically recorded in the analytical database. In patients who developed persistent urinary leakage, retrograde DJ stent placement was performed as a secondary intervention. Once urinary leakage persisted beyond 24 h and met the definition of persistent urinary leakage, retrograde DJ stent placement was performed without further delay, typically within the following few hours. The exact time from secondary DJ stent placement to complete resolution of urinary leakage was not systematically recorded in the analytical database.

No imaging-confirmed urinoma or rehospitalization related to urinary leakage occurred in the study cohort. Apart from the routine plain urinary system radiograph obtained on the first postoperative morning, no additional imaging was routinely performed specifically for persistent urinary leakage during the index hospitalization. Subsequently, in patients without evidence of urinary leakage following nephrostomy tube removal, the Foley catheter was removed together with the ureteral catheter in patients managed with external ureteral drainage, whereas only the Foley catheter was removed in patients who underwent primary DJ stent placement.

At the second postoperative week, a clean-catch midstream urine sample was obtained from all patients for urine culture. All samples were processed using the same standardized microbiological methodology and institutional laboratory protocol. No data were missing for this variable.

Primary DJ stents were routinely left indwelling for approximately 3–4 weeks and were subsequently removed in an outpatient setting under local anesthesia using flexible cystoscopy. Follow-up data after stent removal were not systematically recorded in the analytical database and therefore were not included in the present study. These management protocols were routinely applied to all patients.

Postoperative complications were classified according to the Clavien–Dindo classification. Patients without any postoperative complication were categorized separately as ‘No complication’ and were not included in Grade I. Grade I complications were defined as deviations from the normal postoperative course that did not require pharmacological treatment other than supportive medications such as analgesics, antiemetics, antipyretics, or similar agents. Grade II complications were defined as events requiring pharmacological treatment beyond that permitted for Grade I, including blood transfusion. Grade III complications were defined as complications requiring surgical, endoscopic, or radiological intervention and were further categorized as Grade IIIa when the intervention was performed without general anesthesia and Grade IIIb when general anesthesia was required. Grade IV complications were defined as life-threatening complications requiring intensive care management, and Grade V represented death.

### 2.2. Study Groups

Patients managed with external ureteral-catheter drainage were classified as Group 1 (*n* = 529), whereas patients who underwent primary antegrade DJ stent placement were classified as Group 2 (*n* = 77). Comparative analyses were performed between these two groups regarding demographic characteristics, perioperative findings, and postoperative outcomes. Because treatment allocation was non-randomized, propensity score adjustment was performed to reduce the influence of measured baseline differences between groups.

Additionally, to evaluate factors associated with persistent urinary leakage in patients managed with ureteral catheters, subgroup analyses were performed in Group 1. Patients without urinary leakage were classified as Group A (*n* = 490), while patients who developed urinary leakage were classified as Group B (*n* = 39). Perioperative and postoperative variables potentially associated with urinary leakage development were compared between these subgroups.

### 2.3. Statistical Analysis

Statistical analyses were performed using IBM SPSS Statistics version 27 (IBM Corp., Armonk, NY, USA). Continuous variables were expressed as mean ± standard deviation (SD) or median with interquartile range (IQR), according to their distribution, whereas categorical variables were presented as frequencies and percentages. The distribution of continuous variables was assessed using histograms together with the Kolmogorov–Smirnov and Shapiro–Wilk tests.

Normally distributed continuous variables were compared using Student’s *t*-test and are presented as mean ± SD, whereas non-normally distributed variables were compared using the Mann–Whitney U test and are presented as median (IQR). For non-normally distributed continuous outcomes showing statistically significant between-group differences, the magnitude of the difference was additionally quantified using the Hodges–Lehmann estimator with 95% confidence intervals. Categorical variables were compared using Pearson’s chi-square test or Fisher’s exact/Fisher–Freeman–Halton exact test, as appropriate according to expected cell counts. All statistical tests were two-sided, and a *p* value < 0.05 was considered statistically significant.

Candidate perioperative variables potentially associated with persistent urinary leakage were first evaluated in univariable analyses. Given the limited number of persistent urinary leakage events (*n* = 39), only variables demonstrating a statistically significant association in univariable analyses (*p* < 0.05) were entered into a parsimonious multivariable logistic regression model to minimize the risk of overfitting. Accordingly, nephroscopy time and residual stone status were included in the final model. Multivariable logistic regression analysis was then performed to identify factors independently associated with persistent urinary leakage. Adjusted odds ratios (ORs) with 95% confidence intervals (95% CIs) were reported. The linearity-in-the-logit assumption for nephroscopy time was assessed using the Box–Tidwell approach by including an interaction term between nephroscopy time and its natural logarithm.

Because length of hospital stay showed a right-skewed distribution, it was analyzed using generalized linear models with a gamma distribution and log link. The primary model evaluated the association between postoperative drainage strategy and hospital stay without including persistent urinary leakage, which occurs after drainage allocation and may represent a post-exposure variable. The model was adjusted for age, sex, body mass index, stone burden, stone location, access site, access calyx, number of access tracts, and preoperative serum creatinine. Robust (Huber–White/sandwich) standard errors were used, and exponentiated regression coefficients were reported as mean ratios with 95% confidence intervals. Model fit was assessed using the scaled deviance and scaled Pearson chi-square statistics relative to their degrees of freedom. In a secondary exploratory gamma regression model, persistent urinary leakage was included together with drainage strategy to evaluate its conditional association with hospital stay. Robust (Huber–White/sandwich) standard errors were used. Because persistent urinary leakage occurs after drainage allocation and may lie on the pathway between drainage strategy and hospital stay, this secondary model was not interpreted as estimating the overall association between drainage strategy and hospitalization duration.

To address potential confounding arising from the retrospective, surgeon preference-based allocation of postoperative drainage, propensity scores for primary antegrade double-J stent placement were estimated using multivariable logistic regression. The propensity score model included age, sex, body mass index, stone burden, stone location, access site, access calyx, multiple access tracts, and preoperative serum creatinine. Propensity-score distributions were examined separately in the two drainage groups and summarized using the mean and standard deviation, median and interquartile range, and observed range. The degree of overlap was assessed using the common-support region, defined as the intersection of the observed propensity-score ranges in the two groups, and by calculating the proportion of patients in each group whose propensity scores fell within this region. No patients were excluded on the basis of the common-support assessment. Stabilized inverse probability of treatment weights (IPTW) was subsequently calculated. The distribution of stabilized weights was examined using the minimum and maximum values and selected percentiles (1st, 5th, 25th, 50th, 75th, 95th, and 99th percentiles) to assess the presence of extreme weights. No truncation or trimming of the stabilized weights was applied. Effective sample size after weighting was calculated for the overall cohort and separately for each drainage group. Baseline covariate balance before and after weighting was assessed using absolute standardized mean differences (SMDs), with values <0.10 considered indicative of adequate balance. For multicategorical variables, each category was represented by a binary indicator and category-specific absolute SMDs were calculated; the largest absolute category-specific SMD was used to summarize imbalance for the corresponding variable. Covariate balance before and after IPTW was reported for all variables included in the propensity-score model. The adjusted absolute risk difference in persistent urinary leakage was estimated using an IPTW-weighted linear probability model. The IPTW-adjusted risk difference was calculated as the estimated risk of persistent urinary leakage in the primary DJ-stent group minus that in the ureteral-catheter group. The 95% confidence interval for the adjusted risk difference was obtained using 5000 nonparametric bootstrap resamples, with the propensity-score model and stabilized weights re-estimated within each bootstrap sample to account for uncertainty associated with propensity-score estimation.

Because no urinary leakage events occurred in the primary double-J stent group, conventional maximum-likelihood logistic regression was inappropriate because of complete separation. Therefore, Firth penalized logistic regression was performed as a complementary analysis to obtain finite effect estimates in the presence of separation. Firth penalized logistic regression was performed using the STATS FIRTHLOG extension command (version 1.1.1) in IBM SPSS Statistics version 27.0 (IBM Corp., Armonk, NY, USA), implemented through R version 3.6 with the logistf package (version 1.24). IPTW was used as the primary approach to address measured baseline confounding between the drainage groups, whereas Firth regression specifically addressed the zero-event structure of the outcome and was not intended to provide comprehensive adjustment for confounding. Given the limited number of leakage events, a parsimonious multivariable Firth model was constructed rather than incorporating the full set of propensity score covariates. The model included drainage strategy together with nephroscopy time and residual stone status as key procedural covariates related to urinary leakage. Nephroscopy time was additionally selected because it differed between the drainage groups and showed a significant univariable association with urinary leakage, whereas residual stone status was included because of its strong association with urinary leakage. However, because nephroscopy time included the drainage-placement stage and could therefore partly represent a post-exposure procedural variable, an additional sensitivity analysis was performed using a Firth penalized logistic regression model including drainage strategy and residual stone status but excluding nephroscopy time. Penalized odds ratios, profile-likelihood 95% confidence intervals, and penalized likelihood-ratio *p* values were reported.

## 3. Results

A total of 606 patients undergoing PCNL were included. The mean age was 47.84 ± 13.01 years; 66.8% of the patients were male and 33.2% were female. Baseline demographic, clinical, and perioperative characteristics are presented in Table 1.

### 3.1. Comparison Between Ureteral-Catheter and Primary DJ-Stent Groups

Baseline characteristics of the ureteral catheter and primary double-J stent groups are presented in Table 2. Before weighting, several covariates showed small but potentially relevant between-group imbalances, with absolute SMDs exceeding 0.10. Stabilized IPTW improved balance for most covariates, particularly stone location, access site, and access calyx. After weighting, however, residual imbalance above the prespecified SMD threshold of 0.10 remained for age (SMD = 0.134), body mass index (SMD = 0.119), and preoperative serum creatinine (SMD = 0.119). Complete covariate balance before and after weighting for all variables included in the propensity-score model is presented in Table 3. Propensity-score distributions showed substantial overlap between the two drainage groups. In the ureteral-catheter group, the mean propensity score was 0.122 ± 0.062, with a median of 0.108 (IQR, 0.082–0.142) and an observed range of 0.021–0.470. In the primary DJ-stent group, the corresponding values were 0.164 ± 0.094, 0.136 (IQR, 0.102–0.188), and 0.042–0.471, respectively. The common-support region extended from 0.042 to 0.470 and included 524 of 529 patients (99.1%) in the ureteral-catheter group and 76 of 77 patients (98.7%) in the primary DJ-stent group, indicating substantial overlap between the propensity-score distributions. The stabilized weights had a mean of 1.001 and ranged from 0.270 to 3.038. The 1st, 5th, 25th, 50th, 75th, 95th, and 99th percentiles were 0.380, 0.813, 0.946, 0.977, 1.021, 1.254, and 1.673, respectively. No truncation or trimming of the stabilized weights was applied. The overall effective sample size after weighting was 583.7, compared with the original sample size of 606. The group-specific effective sample sizes were 525.1 for the ureteral-catheter group and 61.8 for the primary DJ-stent group.

Baseline demographic, stone-related, and operative characteristics were comparable between Group 1 and Group 2. There were no statistically significant differences between the groups in terms of age, sex, BMI, stone size, stone localization, access site, access calyx, or number of access tracts (*p* > 0.05).

#### 3.1.1. Intraoperative Outcomes

Intraoperative parameters, including operative time, nephroscopy time, fluoroscopy time, and irrigation fluid volume, were evaluated. Operative time was significantly longer in the primary DJ-stent group compared to the ureteral-catheter group (110.38 ± 47.17 vs. 100.59 ± 37.86 min, *p* = 0.041). Similarly, nephroscopy time was significantly prolonged in the DJ-stent group (*p* = 0.002). In contrast, fluoroscopy time and irrigation fluid volume were comparable between the groups, with no statistically significant differences observed (*p* = 0.447 and *p* = 0.386, respectively) (Table 2).

#### 3.1.2. Postoperative Outcomes

There was no statistically significant difference between the groups in terms of residual stone rates (25.7% vs. 23.4%, *p* = 0.661). Persistent urinary leakage developed in 7.4% of patients in the ureteral-catheter group, whereas no cases of urinary leakage were observed in the primary DJ-stent group (*p* = 0.006). The time to nephrostomy tube removal was significantly shorter in the DJ-stent group (*p* = 0.028). Although the distribution of nephrostomy tube removal time differed significantly between the drainage groups, the absolute difference was modest. The Hodges–Lehmann estimated between-group difference was 0 days (95% CI, 0 to 0). Notably, despite significantly longer operative and nephroscopy times in the primary DJ-stent group, no cases of urinary leakage were observed in these patients. Among patients who developed urinary leakage in the ureteral-catheter group, secondary DJ stent placement was required (*n* = 39).

In the IPTW-adjusted population, the estimated urinary leakage rate was 0% in the primary DJ-stent group and 7.45% in the ureteral-catheter group. The IPTW-adjusted risk difference, calculated as the risk in the primary DJ-stent group minus that in the ureteral-catheter group, was −7.45 percentage points (95% bootstrap CI, −9.77 to −5.33).

Because no urinary leakage events occurred in the primary DJ-stent group, conventional logistic regression was not appropriate because of complete separation. Therefore, Firth penalized logistic regression was performed. In the univariable Firth model, primary DJ stent placement was associated with significantly lower odds of urinary leakage (OR 0.080, 95% CI 0.0006–0.573; *p* = 0.002). In a multivariable Firth penalized logistic regression model additionally including residual stone status and nephroscopy time, the association remained significant (adjusted OR 0.072, 95% CI 0.0006–0.522; *p* = 0.002). Because nephroscopy time included the drainage-placement stage and could partly represent a post-exposure variable, an additional Firth sensitivity analysis was performed excluding nephroscopy time. In this model, the association between primary DJ stenting and persistent urinary leakage remained evident (OR 0.081, 95% profile-likelihood CI 0.0006–0.581; *p* = 0.006), while residual stone presence remained associated with higher odds of persistent urinary leakage (OR 2.42, 95% CI 1.24–4.65; *p* = 0.010).

Hospital stay duration differed significantly between the drainage groups in the unadjusted analysis (median 3.0 [IQR, 3.0–4.0] days in the ureteral-catheter group vs. 3.0 [IQR, 2.0–3.0] days in the primary DJ-stent group; *p* = 0.004). Although the distributions differed significantly, the absolute between-group difference was modest, with a Hodges–Lehmann estimated difference of 0 days (95% CI, 0 to 1). Among patients managed with ureteral catheters, hospital stay was longer in those who developed persistent urinary leakage than in those without leakage (median 4.0 [IQR, 4.0–7.0] vs. 3.0 [IQR, 2.0–4.0] days; *p* < 0.001). Notably, even after excluding patients who developed urinary leakage, the primary DJ-stent group continued to demonstrate a significantly shorter hospitalization duration compared to the ureteral-catheter group (*p* = 0.018). In the primary multivariable gamma regression model with a log link, adjusted for baseline demographic, stone-related, and access-related covariates and excluding persistent urinary leakage, ureteral-catheter drainage was associated with a longer expected hospital stay compared with primary DJ stenting (mean ratio 1.24, 95% CI 1.14–1.35; *p* < 0.001), corresponding to approximately 23.9% longer expected hospitalization. Robust standard errors were used. The scaled deviance/df and scaled Pearson chi-square/df ratios were 1.07 and 1.26, respectively, indicating acceptable model fit. In the secondary exploratory gamma regression model including both drainage strategy and persistent urinary leakage, ureteral-catheter drainage remained associated with a longer expected hospital stay compared with primary DJ stenting (mean ratio 1.18, 95% CI 1.07–1.30; *p* = 0.001). Persistent urinary leakage was also associated with a longer expected hospital stay (mean ratio 1.54, 95% CI 1.35–1.75; *p* < 0.001). Because persistent urinary leakage is a post-exposure variable, this model was interpreted as exploratory and not as an estimate of the overall association between drainage strategy and hospital stay.

There were no significant differences between groups in postoperative fever, sepsis, blood transfusion rates, or postoperative second-week urine culture positivity (*p* > 0.05). (Table 2). No DJ stent-related complications requiring intervention were documented during the index hospitalization.

The distribution of Clavien–Dindo complications did not differ significantly between the drainage groups (*p* = 0.095). In the ureteral-catheter group, 400 patients (75.6%) had no postoperative complication, while Grade I, Grade II, and Grade IIIa complications occurred in 48 (9.1%), 42 (7.9%), and 39 (7.4%) patients, respectively. In the primary DJ-stent group, 62 patients (80.5%) had no postoperative complication, whereas Grade I and Grade II complications occurred in 9 (11.7%) and 6 (7.8%) patients, respectively; no Grade IIIa complications were observed. No Grade IIIb, Grade IV, or Grade V complications occurred in either group. Overall, Grade I events predominantly consisted of minor postoperative symptoms managed with additional analgesic and/or antiemetic therapy. Grade II events consisted of sepsis requiring intravenous antibiotic therapy in 10 patients and blood transfusion in 38 patients, while all 39 Grade IIIa events represented secondary DJ stent placement under local anesthesia for persistent urinary leakage.

### 3.2. Risk Factors for Persistent Urinary Leakage in the Ureteral-Catheter Group

Among patients who initially received a ureteral catheter, a subgroup analysis was performed to identify factors associated with urinary leakage. Stone localization and access site were not significantly associated with urinary leakage development (*p* = 0.067 and *p* = 0.637, respectively). Similarly, the number of access tracts and overall operative time did not differ significantly between patients with and without urinary leakage (*p* = 0.111 and *p* = 0.107, respectively). Access calyx was also not significantly associated with the development of urinary leakage (*p* = 0.053).

Nephroscopy time was significantly longer in patients who developed urinary leakage compared to those who did not (*p* = 0.024). However, in the parsimonious multivariable logistic regression model including nephroscopy time and residual stone status, the association between nephroscopy time and persistent urinary leakage was attenuated and did not retain statistical significance (OR 1.009, 95% CI 0.998–1.020; *p* = 0.100). The Box–Tidwell interaction term for nephroscopy time was not statistically significant (*p* = 0.379), indicating no evidence of violation of the linearity-in-the-logit assumption. When expressed over a clinically interpretable 10-min interval, the adjusted OR was 1.095 (95% CI 0.983–1.220; *p* = 0.100), corresponding to approximately 9.5% higher odds of persistent urinary leakage per 10-min increase in nephroscopy time; however, this association remained statistically non-significant. The amount of irrigation fluid used during the procedure was comparable between the groups (*p* = 0.122).

The presence of residual stones was found to be significantly associated with urinary leakage (43.6% vs. 24.3%, *p* = 0.008), whereas the localization of residual stones did not show a significant relationship (*p* = 0.354). In the multivariable logistic regression model, residual stone presence remained independently associated with persistent urinary leakage and was associated with approximately 2.2-fold higher odds of urinary leakage (OR 2.16, 95% CI 1.09–4.28; *p* = 0.027). The complete results of the parsimonious multivariable logistic regression model are presented in Table 4.

Furthermore, patients who developed urinary leakage had a significantly prolonged hospital stay compared to those without urinary leakage (*p* < 0.001), which was primarily attributed to the need for secondary intervention with DJ stent placement. The perioperative and postoperative factors associated with urinary leakage among patients who initially underwent ureteral catheter placement are presented in Table 5.

## 4. Discussion

Although postoperative drainage following PCNL has been investigated previously, most comparative studies have primarily focused on drainage techniques or tubeless PCNL protocols. Likewise, studies evaluating predictors of persistent postoperative urinary leakage have largely been conducted in cohorts managed with a single postoperative drainage strategy, predominantly external ureteral catheterization, without specifically addressing the potential influence of drainage modality itself [7]. In contrast, the present study evaluated postoperative drainage strategy in relation to persistent postoperative urinary leakage and early postoperative recovery in a large retrospective cohort of patients undergoing conventional nephrostomy-tube PCNL, including patients managed with either an external ureteral catheter or a primary antegrade DJ stent.

The principal findings of this study were that postoperative drainage strategy was associated with persistent postoperative urinary leakage, nephrostomy tube removal time, and length of hospital stay. Furthermore, among patients managed with external ureteral catheters, longer nephroscopy time was associated with persistent postoperative urinary leakage in univariable analysis; however, this association was attenuated and did not remain statistically significant after multivariable adjustment. In contrast, residual stone presence remained independently associated with persistent urinary leakage. Together, these findings provide additional insight into factors associated with early postoperative recovery after conventional PCNL and may contribute to postoperative drainage decision-making in routine clinical practice.

### 4.1. Intraoperative Outcomes

The significantly longer operative and nephroscopy times observed in the primary DJ-stent group in our study may be attributed to the additional procedural steps required for antegrade DJ stent placement at the end of the operation. These additional manipulations inevitably prolong both operative and nephroscopy times. Supporting our findings, Falahatkar et al. reported significantly longer operative times in patients undergoing supine PCNL with postoperative DJ stent placement in a cross-sectional study evaluating staghorn stones [9]. Similarly, in a retrospective comparative study, Gönülalan et al. divided PCNL patients into three groups according to postoperative drainage method—nephrostomy tube only, DJ stent only, and ureteral catheter only—and demonstrated that the shortest operative times were observed in the ureteral-catheter group [10]. Comparable trends favoring ureteral catheter placement in terms of shorter operative duration have also been reported in other retrospective studies, although these differences did not always reach statistical significance [11,12]. In contrast, several randomized controlled trials have demonstrated no significant increase in operative time associated with DJ stent placement during PCNL [13,14,15]. Likewise, two recent meta-analyses evaluating drainage strategies after PCNL reported shorter operative times in ureteral-catheter groups compared to DJ-stent groups; however, these findings were not statistically significant [16,17].

Nevertheless, it should be emphasized that the available literature is highly heterogeneous, with most published studies focusing predominantly on tubeless PCNL approaches. In contrast, all patients in our cohort routinely underwent nephrostomy tube placement in addition to either a DJ stent or ureteral catheter according to our institutional protocol. Another important consideration is that multiple techniques for antegrade DJ stent placement have been described in the literature, and the preferred method may vary considerably between institutions and surgeons [18]. Such technical variations may substantially influence perioperative parameters including operative and nephroscopy times. Therefore, further prospective studies with larger patient populations and standardized stenting techniques are warranted.

Although primary DJ stent placement was associated with longer operative and nephroscopy times in our study, total fluoroscopy duration did not differ significantly between the groups. Direct comparative PCNL studies specifically quantifying fluoroscopy exposure attributable to antegrade stent placement remain limited. Nevertheless, this finding is mechanistically plausible because the majority of fluoroscopy exposure during PCNL occurs during calyceal access and tract dilation, whereas end-of-procedure DJ stent placement generally requires only brief confirmatory fluoroscopic imaging rather than prolonged continuous fluoroscopic guidance. This interpretation is supported by minimal-fluoroscopy endourological series demonstrating that stent confirmation contributes minimally to overall fluoroscopic exposure, as well as PCNL studies reporting substantial variability in fluoroscopy duration independent of operative time [19,20].

### 4.2. Postoperative Drainage and Persistent Urinary Leakage

In the present study, the distribution of nephrostomy tube removal time differed significantly between the drainage groups, favoring the DJ-stent group. However, the absolute magnitude of this difference was small, with a Hodges–Lehmann estimated between-group difference of 0 days (95% CI, 0 to 0), indicating that the statistically significant difference should be interpreted cautiously in terms of clinical magnitude. This finding is physiologically expected, as internal ureteral drainage provided by the DJ stent facilitates continuous antegrade urinary flow from the collecting system to the bladder, thereby reducing intrapelvic pressure and promoting more effective urinary drainage during the postoperative period [21]. Improved internal drainage may also minimize urinary extravasation through the nephrostomy tract and reduce the impact of transient ureteral edema following endourological manipulation, ultimately allowing earlier nephrostomy tube removal [22,23]. Accordingly, we believe that once the nephrostomy tube was removed, the presence of a DJ stent enabled rapid restoration of antegrade urinary flow into the bladder, thereby preventing symptoms related to urinary leakage from the nephrostomy tract or obstruction-induced acute pain. These mechanisms may have contributed to the observed difference in the distribution of nephrostomy tube removal time between the groups. Similar to our findings, previous studies have demonstrated that DJ stent placement during PCNL reduces the incidence of postoperative urinary leakage [24].

Conversely, the distribution of nephrostomy tube removal times in the ureteral-catheter group was shifted toward later removal, although the absolute between-group difference remained small. Although previous studies have demonstrated that short-distance mobilization of up to 5 m does not necessarily result in distal migration of ureteral catheters below the ureteropelvic junction, ureteral catheters may theoretically fail to maintain optimal positioning within the renal pelvis and may migrate into the ureter during patient mobilization, thereby compromising adequate drainage [25]. Even when initially positioned appropriately, catheter displacement may impair continuous urinary flow and reduce drainage efficiency compared to an internally fixed DJ stent. Consistent with this hypothesis, urinary leakage was significantly more frequent in the ureteral-catheter group in our study. Previous reports have similarly demonstrated higher urinary leakage rates associated with ureteral catheter drainage strategies [26]. However, in a randomized controlled trial, although urinary leakage rates were numerically higher in the ureteral-catheter group compared to the DJ-stent group, this difference did not reach statistical significance [27]. Further studies comprehensively evaluating variables such as ureteral catheter length, diameter, and precise positioning of the proximal tip within the pelvicalyceal system are needed.

The association between primary DJ stent placement and lower urinary leakage occurrence remained evident after adjustment for measured confounding. Stabilized inverse probability weighting demonstrated a lower estimated risk of urinary leakage in the primary DJ-stent group, reflected by an IPTW-adjusted risk difference of −7.45 percentage points. However, IPTW does not generate outcome events in a treatment group in which none were observed; therefore, the IPTW-adjusted risk-difference estimate remains dependent on the zero-event structure of the primary DJ-stent group and should be interpreted with appropriate caution. Firth penalized logistic regression produced a finite effect estimate despite the absence of events in the DJ-stent group. Nevertheless, the wide confidence interval reflects the limited number of patients receiving primary DJ stents and the zero-event structure of the outcome. Residual confounding related to surgeon preference and unmeasured procedural factors cannot be excluded.

Importantly, all Grade IIIa complications represented secondary DJ stent placement for persistent urinary leakage. Therefore, the occurrence of Grade IIIa complications was directly linked to the primary outcome and should not be interpreted as independent confirmation of an overall safety advantage of primary DJ stenting.

### 4.3. Length of Hospital Stay

The distribution of hospital stay differed significantly between the drainage groups, favoring patients who received primary DJ stent placement. However, the absolute magnitude of this difference was modest, with a Hodges–Lehmann estimated between-group difference of 0 days (95% CI, 0 to 1 day), indicating that the statistical difference should be interpreted cautiously in terms of clinical relevance. In the primary gamma regression model excluding persistent urinary leakage and adjusting for measured baseline covariates, ureteral-catheter drainage remained associated with a longer expected hospital stay compared with primary DJ stenting (mean ratio 1.24, 95% CI 1.14–1.35; *p* < 0.001). In the secondary exploratory model, inclusion of persistent urinary leakage attenuated but did not eliminate the association between drainage strategy and hospitalization duration (mean ratio 1.18, 95% CI 1.07–1.30; *p* = 0.001), while persistent urinary leakage itself was associated with longer expected hospitalization (mean ratio 1.54, 95% CI 1.35–1.75; *p* < 0.001). However, because persistent urinary leakage occurs after drainage allocation and may lie on the pathway between drainage strategy and hospital stay, the model including leakage should not be interpreted as estimating the overall association of drainage strategy with hospitalization duration. We believe that earlier nephrostomy tube removal and the reduced need for secondary interventions due to urinary leakage contributed substantially to this finding. The persistence of an association between drainage strategy and hospital stay in the primary model, in which postoperative urinary leakage was not included, suggests that the observed difference in hospitalization duration was not solely attributable to conditioning on urinary leakage. We speculate that this finding may be related to differences in postoperative urinary drainage dynamics. During nephrostomy tube clamping trials, antegrade urinary flow from the kidney to the bladder is likely established more efficiently in patients with a DJ stent. In contrast, some patients managed with ureteral catheters may experience partial transient obstruction caused by ureteral edema, blood clots, residual fragments, or suboptimal catheter positioning. Following nephrostomy tube clamping, such conditions may result in flank pain secondary to impaired urinary drainage, necessitating reopening of the nephrostomy tube and delaying subsequent removal attempts. These mechanisms may therefore have contributed to the observed distributional difference in hospital stay, although the absolute between-group difference was small. Consequently, these events may contribute to prolonged hospitalization in patients managed with ureteral catheters, even in the absence of clinically evident urinary leakage. Supporting our results, Agrawal et al. similarly reported lower urinary leakage and secondary intervention rates, as well as shorter hospitalization times, in patients receiving DJ stents [24]. However, unlike our study, nephrostomy tubes were not routinely placed in the DJ-stent group in that study. Conversely, other studies have suggested that DJ stent placement does not significantly reduce hospital stay duration [14,27]. Importantly, the majority of published studies in this field have focused on tubeless PCNL, whereas our study differs by evaluating patients who routinely underwent nephrostomy tube placement in addition to either ureteral catheterization or DJ stenting.

### 4.4. Predictors of Persistent Urinary Leakage

When factors associated with persistent urinary leakage in the ureteral-catheter group were evaluated, longer nephroscopy time and residual stone presence were significantly associated with urinary leakage in univariable analyses. Prolonged nephroscopy duration has previously been reported to be associated with postoperative urinary leakage [7]. In the present study, however, the association between nephroscopy time and persistent urinary leakage was attenuated after adjustment for residual stone status and did not retain statistical significance in the multivariable model. Therefore, although the direction of the association is consistent with previous observations, nephroscopy duration cannot be considered an independently associated factor in our cohort.

Interestingly, despite significantly longer nephroscopy times in the primary DJ-stent group, no urinary leakage events were observed in these patients. This observation may be compatible with a possible role of postoperative drainage strategy in modifying leakage occurrence; however, it should be interpreted cautiously, particularly because nephroscopy time did not remain significantly associated with urinary leakage after multivariable adjustment, and because drainage allocation was non-randomized with no leakage events observed in the DJ-stent group. Residual stone presence, in contrast, remained independently associated with higher odds of persistent urinary leakage in the multivariable analysis. We speculate that residual fragments may contribute to partial obstruction proximal to the ureteral catheter tip, thereby impairing urinary drainage and facilitating urinary extravasation.

### 4.5. Limitations

This study has several limitations. First, its retrospective single-center design may have introduced selection bias and limited control over potential confounding variables. In addition, although all participating surgeons were experienced in PCNL, the choice between ureteral catheter placement and primary DJ stent insertion was based on individual surgeon preference and intraoperative judgment rather than randomization or standardized selection criteria, which may have affected patient allocation and perioperative decision-making. Moreover, the specific intraoperative reasons underlying the surgeon’s decision to place a primary DJ stent were not systematically evaluated in the present study.

An additional major limitation is that patient-level operating-surgeon identifiers and reliable calendar-year or operative-period information were not available in the analyzable database and could not be reconstructed with sufficient accuracy. Consequently, the distribution of drainage strategies according to surgeon or calendar period could not be presented, and surgeon-level adjustment or clustering and calendar-time adjustment could not be incorporated into the propensity-score model or examined in sensitivity analyses. Because drainage selection was influenced by individual surgeon preference and intraoperative judgment, and perioperative and postoperative practices may also have varied across surgeons or over the extended study period, residual confounding related to inter-surgeon and temporal differences cannot be excluded. Therefore, some of the observed differences in postoperative management and length of hospital stay may partly reflect these unmeasured surgeon- or time-related factors rather than drainage strategy alone. The marked imbalance in group sizes between the ureteral-catheter and primary DJ-stent groups may have affected the precision of between-group comparisons, particularly for outcomes with low event rates. Although adjusted analyses were performed to reduce the impact of measured confounding, this imbalance should be considered when interpreting the findings.

Furthermore, the relatively limited number of urinary leakage events may have reduced the statistical power of subgroup and multivariate analyses. Additionally, patients who experienced major intraoperative or perioperative complications requiring substantial deviation from the standard postoperative management pathway were excluded. Although this approach was intended to minimize confounding from complication-driven postoperative management, such exclusion may have introduced additional selection bias and may limit the generalizability of our findings to patients without major perioperative events.

Another important limitation is that tract length and renal parenchymal thickness, both of which may influence postoperative urinary leakage, were not evaluated. Previous studies have demonstrated that these anatomical factors may directly affect the risk of urinary extravasation following PCNL [7,28]. In addition, intrarenal pelvic pressure was not measured intraoperatively, and postoperative positional changes or migration of ureteral catheters were not objectively assessed radiologically. Although all patients remained hospitalized for more than 24 h after nephrostomy tube removal, delayed or recurrent urinary leakage occurring after discharge was not systematically captured and may therefore have gone unrecorded. In addition, the urinary origin of dressing wetness was not biochemically confirmed, introducing a potential source of outcome misclassification. Leakage assessment was based on non-blinded clinical observation, leakage volume was not quantitatively measured, and the exact duration of leakage beyond the predefined 24-h threshold was not systematically recorded.

Finally, DJ stent-related symptoms such as flank pain, dysuria, urinary urgency, and irritative lower urinary tract symptoms were not evaluated. Considering that DJ stents remained indwelling for approximately 3–4 weeks in our cohort, these symptoms may represent a significant source of postoperative morbidity and may lead to additional outpatient visits or requests for early stent removal [27,29]. In addition, unscheduled healthcare visits, readmissions, post-discharge infections, and the costs and potential complications associated with subsequent stent removal were not systematically assessed. Therefore, the present study primarily reflects early inpatient outcomes and does not provide a comprehensive assessment of the overall benefit–risk balance of primary DJ stenting.

## 5. Conclusions

In this single-center, retrospective cohort study, primary antegrade DJ stenting was associated with a lower incidence of persistent urinary leakage and slightly faster early postoperative recovery compared with external ureteral catheterization. However, the non-randomized choice of drainage, the relatively small primary DJ-stent group, the limited number of leakage events, the absence of leakage events in the DJ-stent group, and the likelihood of residual confounding do not allow us to conclude a causal or universal advantage of this method. These findings require confirmation in prospective randomized trials that also evaluate stent-related complications and symptoms.

## Figures and Tables

**Table 1:** Baseline characteristics of patients undergoing prone percutaneous nephrolithotomy.

Age (years)		47.84 ± 13.01
Sex, n (%)	Female	201 (33.20%)
	Male	405 (66.80%)
Body mass index (kg/m^2^)		26.57 ± 4.53
Stone location, n (%)	Lower calyx	58 (9.60%)
	Middle calyx	13 (2.10%)
	Upper calyx	11 (1.80%)
	Renal pelvis	139 (22.90%)
	Staghorn stone	77 (12.70%)
	Proximal ureter	16 (2.60%)
	Partial staghorn stone	97 (16.00%)
	Pelvis + calyx	174 (28.70%)
	Multiple calyces	21 (3.50%)
Stone burden (mm^2^) median (IQR)		314.00 (212.00–544.00)
Preoperative serum creatinine (mg/dL)		1.01 ± 0.51
Preoperative eGFR (mL/min/1.73 m^2^)		100.70 ± 31.22
Access site, n (%)	Subcostal	383 (63.20%)
	Intercostal (11th–12th rib)	202 (33.30%)
	Combined intercostal and subcostal	15 (2.50%)
	Intercostal (10th–11th rib)	6 (1.00%)
Access calyx, n (%)	Upper calyx	23 (3.80%)
	Middle calyx	186 (30.70%)
	Lower calyx	360 (59.40%)
	Multiple calyces	37 (6.10%)
Number of access tracts, n (%)	Single tract	551 (90.90%)
	Multiple tracts	55 (9.10%)
Operative time (min)		101.83 ± 39.25
Nephroscopy time (min)		45.97 ± 26.31
Fluoroscopy time (s)		79.09 ± 59.67
Clavien–Dindo grade, n (%)	No complication	462 (76.2%)
	Grade I	57 (9.4%)
	Grade II	48 (7.90%)
	Grade IIIa	39 (6.40%)
	Grade IIIb	0 (0.00%)
	Grade IV	0 (0.00%)
	Grade V	0 (0.00%)
Postoperative fever, n (%)	Present	31 (5.10%)
	Absent	575 (94.90%)
Postoperative sepsis, n (%)	Present	10 (1.70%)
	Absent	596 (98.30%)
Blood transfusion, n (%)	Present	38 (6.30%)
	Absent	568 (93.70%)
Length of hospital stay median (IQR) (days)		3.00 (2.00–4.00)

IQR, interquartile range; BMI, body mass index; eGFR, estimated glomerular filtration rate.

**Table 2:** Baseline characteristics and perioperative and postoperative outcomes according to drainage strategy.

		Group 1 (Ureteral Catheter)	Group 2 (Primary DJ Stent)	*p* Value
**Baseline** **characteristics**				
Age (years)		48.04 ± 12.74	46.51 ± 14.79	0.338 ^Δ^
Sex	Female	174 (32.90%)	27 (35.10%)	0.705 ^α^
	Male	355 (67.10%)	50 (64.90%)	
Body mass index (kg/m^2^)		26.47 ± 4.55	27.10 ± 4.40	0.256 ^Δ^
Stone location	Lower Calyx	53 (10.00%)	5 (6.50%)	0.133 ^β^
	Middle Calyx	12 (2.30%)	1 (1.30%)	
	Upper Calyx	10 (1.90%)	1 (1.30%)	
	Renal Pelvis	126 (23.80%)	13 (16.90%)	
	Staghorn Stone	64 (12.10%)	13 (16.90%)	
	Proximal Ureter	10 (1.90%)	6 (7.80%)	
	Partial Staghorn Stone	86 (16.30%)	11 (14.30%)	
	Pelvis + Calyx	151 (28.50%)	23 (29.90%)	
	Multiple Calyces	17 (3.20%)	4 (5.20%)	
Stone burden (mm^2^) median (IQR)		319.00 (205.00–534.50)	314.00 (223.00–571.00)	0.936 ^Σ^
Access site	Subcostal	331(62.60%)	52 (67.50%)	0.813 ^α^
	Intercostal (11th–12th Rib)	180 (34.00%)	22 (28.60%)	
	Combined Intercostal and Subcostal	13 (2.50%)	2 (2.60%)	
	Intercostal (10th–11th Rib)	5 (0.90%)	1 (1.30%)	
Access calyx	Upper Calyx	18 (3.40%)	5 (6.50%)	0.510 ^β^
	Middle Calyx	165 (31.20%)	21 (27.30%)	
	Lower Calyx	313 (59.20%)	47 (61.00%)	
	Multiple Calyces	33 (6.20%)	4 (5.20%)	
Number of access tracts	Single Tract	480 (90.70%)	71 (92.20%)	0.446 ^α^
	Multiple Tracts	49 (9.30%)	6 (7.80%)	
**Intraoperative outcomes**				
Operative time (min)		100.59 ± 37.86	110.38 ± 47.17	0.041 ^Δ^
Nephroscopy time (min), median (IQR)		40.00 (30.00–60.00)	50.00 (30.00–60.00)	0.002 ^Σ^
Fluoroscopy time (s), median (IQR)		65.00 (41.50–99.50)	64.00 (39.50–96.50)	0.447 ^Σ^
Irrigation fluid volume (mL)		12,000.00 (9000.00–18,000.00)	12,000.00 (9000.00–18,000.00)	0.386 ^Σ^
**Postoperative outcomes**				
Residual stone *	Absent	393 (74.30%)	59 (76.60%)	0.661 ^α^
	Present	136 (25.70%)	18 (23.40%)	
Clavien–Dindo classification	No complication	400 (75.60%)	62 (80.50%)	0.095 ^α^
	Grade I	48 (9.10%)	9 (11.70%)	
	Grade II	42 (7.90%)	6 (7.80%)	
	Grade IIIa	39 (7.40%)	0 (0.00%)	
	Grade IIIb	0 (0.00%)	0 (0.00%)	
	Grade IV	0 (0.00%)	0 (0.00%)	
	Grade V	0 (0.00%)	0 (0.00%)	
Blood transfusion	No	496 (93.80%)	72 (93.50%)	0.931 ^α^
	Yes	33 (6.20%)	5 (6.50%)	
Time to nephrostomy tube removal (days)		2.00 (2.00–2.50)	2.00 (2.00–2.00)	0.028 ^Σ^
Persistent urinary leakage, n (%)	No	490 (92.60%)	77 (100.0%)	0.006 ^β^
	Yes	39 (7.40%)	0 (0.00%)	
Postoperative fever, n (%)	No	503 (95.10%)	72 (93.50%)	0.557 ^α^
	Yes	26 (4.90%)	5 (6.50%)	
Postoperative sepsis, n (%)	No	520 (98.30%)	76 (98.70%)	0.796 ^α^
	Yes	9 (1.70%)	1 (1.30%)	
Length of hospital stay, median (IQR) (days)		3.00 (3.00–4.00)	3.00 (2.00–3.00)	0.004 ^Σ^
Length of hospital stay excluding patients with persistent urinary leakage, median (IQR) (days)		3.00 (2.00–4.00)	3.00 (2.00–3.00)	0.018 ^Σ^
Positive urine culture at postoperative week 2, n (%)	No	484 (91.50%)	68 (88.30%)	0.360 ^α^
	Yes	45 (8.50%)	9 (11.70%)	

IQR: interquartile range; ^Σ^: Mann–Whitney U test; ^Δ^: Student’s *T* test; ^α^: Chi-square test; ^β^: Fisher’s exact test/Fisher–Freeman–Halton exact test. BMI, body mass index; eGFR, estimated glomerular filtration rate; DJ, double-J. * Based on end-of-procedure fluoroscopic/nephroscopic assessment and postoperative-day-1 plain kidney–ureter–bladder radiography.

**Table 3:** Covariate balance before and after stabilized inverse probability of treatment weighting.

	Absolute SMD Before IPTW	Absolute SMD After IPTW
Age	0.111	0.134
Sex	0.046	0.012
Body mass index	0.141	0.119
Stone burden	0.004	0.009
Stone location ^†^	0.278	0.093
Access site ^†^	0.118	0.012
Access calyx ^†^	0.143	0.042
Multiple access tracts	0.053	0.006
Preoperative serum creatinine	0.091	0.119

IPTW: inverse probability of treatment weighting; SMD: standardized mean difference. ^†^ For multicategorical variables, each category was represented by a binary indicator and category-specific absolute SMDs were calculated; the largest absolute category-specific SMD is reported for the corresponding variable. An absolute SMD < 0.10 was considered indicative of adequate balance.

**Table 4:** Multivariable logistic regression analysis of factors associated with persistent urinary leakage among patients managed with ureteral catheters.

	B	S.E.	Adjusted OR	95% CI for OR	*p* Value
Nephroscopy time, per 10-min increase	0.091	0.055	1.095	0.983–1.220	0.100
Residual stone presence (vs. absence)	0.770	0.349	2.160	1.090–4.280	0.027

OR: odds ratio; CI: confidence interval; S.E.: standard error.

**Table 5:** Perioperative and postoperative factors associated with persistent urinary leakage in patients managed with ureteral catheters.

		Group A (No Urinary Leakage)	Group B (Urinary Leakage)	*p* Value
Stone Location	Lower Calyx	52 (10.60%)	1 (2.60%)	0.067 ^α^
	Middle Calyx	10 (2.00%)	2 (5.10%)	
	Upper Calyx	8 (1.60%)	2 (5.10%)	
	Renal Pelvis	118 (24.10%)	8 (20.50%)	
	Staghorn Stone	54 (11.00%)	10 (25.60%)	
	Proximal Ureter	10 (2.00%)	0 (0.00%)	
	Partial Staghorn Stone	79 (16.10%)	7 (17.90%)	
	Pelvis + Calyx	143 (29.20%)	8 (20.50%)	
	Multiple Calyces	16 (3.30%)	1 (2.60%)	
Access Site	Subcostal	305 (62.20%)	26 (66.70%)	0.637 ^α^
	Intercostal (11th–12th Rib)	169 (34.50%)	11 (28.20%)	
	Combined Intercostal and Subcostal	12 (2.40%)	1 (2.60%)	
	Intercostal (10th–11th Rib)	4 (0.80%)	1 (2.60%)	
Access Calyx	Upper Calyx	14 (2.90%)	4 (10.30%)	0.053 ^β^
	Middle Calyx	156 (31.80%)	9 (23.10%)	
	Lower Calyx	291 (59.40%)	22 (56.40%)	
	Multiple Calyces	29 (5.90%)	4 (10.30%)	
Number of Access Tracts	Single Tract	448 (91.4%)	32 (82.1%)	0.111 ^α^
	Multiple Tracts	42 (8.6%)	7 (17.9%)	
Operative Time (min)		99.84 ± 37.04	110.00 ± 46.49	0.107 ^Δ^
Nephroscopy Time (min), median (IQR)		40.00 (30.00–55.00)	50.00 (30.00–65.00)	0.024 ^Σ^
Irrigation Fluid Volume (mL), median (IQR)		12,000.00 (8000.00–18,000.00)	14,000.00 (10,000.00–24,000.00)	0.122 ^Σ^
Residual Stone	No	371 (75.70%)	22 (56.40%)	0.008 ^α^
	Yes	119 (24.30%)	17 (43.60%)	
Residual Stone Location	Lower Pole	73 (61.34%)	13 (76.47%)	0.354 ^α^
	Middle Pole	20 (16.81%)	3 (17.65%)	
	Upper Pole	14 (11.76%)	0 (0.00%)	
	Renal Pelvis	3 (2.52%)	1 (5.88%)	
	Multiple Locations	9 (7.56%)	0 (0.00%)	
Length of Hospital Stay, median (IQR) (days)		3.00 (2.00–4.00)	4.00 (4.00–7.00)	<0.001 ^Σ^

IQR: interquartile range; ^Σ^: Mann–Whitney U test; ^Δ^: Student’s *T* test; ^α^: Chi-square test; ^β^: Fisher’s exact test/Fisher–Freeman–Halton exact test.

## Data Availability

The raw data supporting the conclusions of this article will be made available by the authors on request.

## Abbreviations

The following abbreviations are used in this manuscript:

BMI	Body mass index
CI	Confidence interval
CT	Computed tomography
DJ	Double-J
eGFR	Estimated glomerular filtration rate
IQR	Interquartile range
IPTW	Inverse probability of treatment weighting
OR	Odds ratio
PCNL	Percutaneous nephrolithotomy
SD	Standard deviation
SMD	Standardized mean difference
